# IoT Operating System Based Fuzzy Inference System for Home Energy Management System in Smart Buildings

**DOI:** 10.3390/s18092802

**Published:** 2018-08-25

**Authors:** Qurat-ul Ain, Sohail Iqbal, Safdar Abbas Khan, Asad Waqar Malik, Iftikhar Ahmad, Nadeem Javaid

**Affiliations:** 1 School of Electrical Engineering and Computer Science (SEECS), National University of Sciences and Technology (NUST), Islamabad 44000, Pakistan; qain.mscs16seecs@seecs.edu.pk (Q.-u.A.); safdar.abbas@seecs.nust.edu.pk (S.A.K.); asad.malik@seecs.edu.pk (A.W.M.); iftikhar.rana@seecs.edu.pk (I.A.); 2 COMSATS Institute of Information Technology, Islamabad 44000, Pakistan; nadeemjavaidqau@gmail.com

**Keywords:** home energy management system, demand-side management, time-of-use, fuzzy logic, user comfort

## Abstract

Energy consumption in the residential sector is 25% of all the sectors. The advent of smart appliances and intelligent sensors have increased the realization of home energy management systems. Acquiring balance between energy consumption and user comfort is in the spotlight when the performance of the smart home is evaluated. Appliances of heating, ventilation and air conditioning constitute up to 64% of energy consumption in residential buildings. A number of research works have shown that fuzzy logic system integrated with other techniques is used with the main objective of energy consumption minimization. However, user comfort is often sacrificed in these techniques. In this paper, we have proposed a Fuzzy Inference System (FIS) that uses humidity as an additional input parameter in order to maintain the thermostat set-points according to user comfort. Additionally, we have used indoor room temperature variation as a feedback to proposed FIS in order to get the better energy consumption. As the number of rules increase, the task of defining them in FIS becomes time consuming and eventually increases the chance of manual errors. We have also proposed the automatic rule base generation using the combinatorial method. The proposed techniques are evaluated using Mamdani FIS and Sugeno FIS. The proposed method provides a flexible and energy efficient decision-making system that maintains the user thermal comfort with the help of intelligent sensors. The proposed FIS system requires less memory and low processing power along with the use of sensors, making it possible to be used in the IoT operating system e.g., RIOT. Simulation results validate that the proposed technique reduces energy consumption by 28%.

## 1. Introduction

During recent decades, concerns over managing energy consumption are rising significantly. Among all the energy consuming economic sectors, namely industrial, transportation, residential and commercial, the residential sector is the third highest energy consumer [1]. The residential sector is responsible for approximately 21% and 17% of total energy consumption in U.S.A., and Canada, respectively [2]. As the total population of the world is increasing swiftly, electricity demand is also estimated to increase by 24% by 2035 [3]. Among all the residential appliances, Heating, Ventilation and Air Conditioning (HVAC) is the main component for user comfort and target for energy consumption minimization as these appliances constitute the major part of residential energy consumption [4]. HVAC appliances are the main electrical loads observed during peak hours [5].

In order to manage the energy consumption, smart home with its Home Energy Management System (HEMS) plays an important role by improving the efficiency, reliability and the conservation of energy usage. A set of incentives and initiatives such as Demand Response (DR), Energy Efficiency (EE), Time of Use (ToU) rates, and Real Time Pricing (RTP), etc. have been introduced by the utilities and smart grid to encourage the energy consumer for participating in load management techniques [6,7]. However, it is often observed that success of these initiatives highly depends on the user acceptance and participation [8]. Demand response and user participation can be facilitated by the use of a thermostat, electric water heater, etc., so that users can program and schedule their devices accordingly [9,10]. HEMS respond to these varying prices by either scheduling or curtailing the load.

With the advancement of embedded devices, intelligent sensors and availability of the Internet, Internet of Things (IoT) has proved its usefulness in the energy conservation domain. Sensors are being used to monitor different environmental condition variations and help to manage the growing number of appliances in an automatic way and maintain the comfortable environment. IoT has found its way to different devices in homes like air conditioning, lighting, etc., and these appliances are connected to the Internet so that these devices can be controlled remotely using smart phones. With the increase in the number of home appliances and sensors, cost and complexity of processing is increasing day by day. The operating system provides a link between software and hardware. When IoT is considered, it brings its own set of constraints for the operating system in terms of memory, size, power and processing capacity. Because of the variation in appliances and connection in IoT, there are various operating systems available in the market, e.g., Real Time Operating System (RTOS), TinyOS, Real-time Internet of Things (RIOT), Android Things, etc. IoT enhances the smart control of HEMS by using sensors, actuators and controllers. However, energy consumption minimization, user comfort enhancement, cost reduction and Peak-to-Average Ratio (PAR) remain major challenges of the HEMS.

Different novel appliance scheduling schemes [11] based on linear programming [12], particle swarm optimization [13], linear sequential multiloop algorithm [14], game theory [15], dynamic programming [16] and fuzzy logic system [17] have been proposed for residential energy optimization and management. Hierarchical control of Alternating Current (AC) and Direct Current (DC) micro-grid has been discussed in [18], whereas decentralized autonomous management techniques [19,20] for independent control and agent-based modelling in order to optimize the individual stakeholder objectives [21] have been proposed in previous research. In order to get the benefit of the demand-side management techniques, a fully neuron connected network of micro-grid [22], particle swarm optimization [23] and linear programming in combination with fuzzy logic [24] have been proposed.

A nonlinear sliding mode controller has been proposed in [25] by coupling humidity and temperature for energy efficient working of HVAC. The proposed technique has surpassed the conventional PID controller in terms of steady state, settling and rise time. The performance of model predictive control has been compared to the PID controllers as well. In [26], the model of a house considering climatic data of a Portuguese city for the functioning of an air conditioner has been simulated for the demand response. Six ToU rates have been considered and an optimal solution was selected on the basis of expenses incurred. Similar research has been conducted by the same authors in [27] while considering the thermal comfort.

However, it has been seen that previous efforts in energy optimization often results in jeopardizing the user comfort as the major focus of these studies were mainly on energy consumption minimization at the cost of user comfort. Recent research work on an energy management system for residential buildings is concentrated on the scheduling of appliances to off-peak hours, which can cause delay in working of appliances and increase of PAR. These approaches are also non-adaptive according to the user schedule.

There are different environment variables that constitute the user comfort e.g., indoor temperature, outdoor temperature, humidity, etc. Therefore, in order to efficiently utilize the benefits of DR incentive while maintaining user comfort, there is a need to consider more parameters that directly affect the user. Our proposed methodology is considering the objectives of user thermal comfort maintenance along with energy consumption minimization, which were not considered in the previous studies [17,28]. Using a humidity parameter allows us to set the thermostat set-points that not only lie in the resident’s comfort zone but also result in energy conservation. In order to evaluate the energy consumption of HVAC for all the possible scenarios that occur due to considered input parameters, a large number of rules to be defined in an FIS rule base pose a difficulty. In this paper, a method is proposed that uses all of the combinations of the input parameters membership function and define all the rules in a less cumbersome manner. In addition to this, a feedback loop is added to the proposed FIS, which constantly checks the change in the indoor room temperature and controls the working of the heater specifically. The proposed system in this paper requires less memory to evaluate the rules in the FIS rule base (approximately 2 kB). The proposed fuzzy controller also requires less power and makes it possible for the controller to be integrated at the home level as a working mechanism using RIOT.

The remainder of paper is structured as follows: state-of-the-art work is categorized and tabulated in Section 2; Section 3 elaborates the problem formulation; in Section 4, the system model based on the fuzzy logic system is presented; simulation results in Section 5 is followed by Section 6 where conclusion of the paper is discussed. Table 1 describes the variables and the abbreviations used throughout this paper.

## 2. Related Work

When dealing with HEMS, researchers have come to find that electric load can be categorized as thermostatically controlled and manually controlled. HVAC devices like air conditioners, heaters, and heat pumps are thermostatically controlled loads. HVAC devices are the major contributors of peak load during peak hours. Load shedding and scheduling of these devices can result in energy consumption minimization. The literature includes a wide range of work related to scheduling and load shedding of different home appliances.

### 2.1. Nature-Based Algorithms for DSM

In the literature, it is observed that nature-based algorithms are often used for scheduling the appliances [29] as nature has provided a beautiful source to researchers for solving complicated problems from different research and problem domains.

A mathematical Wind Driven Optimization (WDO) [30] technique has been used to achieve user comfort in terms of appliance waiting time and objective of electricity cost minimization. Appliances were divided into three different classes and the hybrid of WDO along with the Knapsack Problem (K-WDO) has been used. Simulations were performed in comparison with Particle Swarm Optimization (PSO), where the proposed technique results in better cost reduction.

In order to increase the user convenience in residential buildings, an approach of the home energy management based on neural network and Q-learning algorithm has been proposed [31]. Results have shown that the technique not only reduced the peak formation and resulted in energy conservation but also helped in reducing carbon footprints. A novel air conditioning system [32] has been developed for better demand response by shifting the load in order to balance the power of the smart grid. The proposed system used two demand response strategies named Demand-Side Bidding (DSB) strategy and Demand as Frequency-controlled Reserve (DFR) strategy. The objective of the study was cost and energy saving while considering a building in Hong Kong with the synthetic dynamic price for RTP. Simulations have been performed for the 40-story office building using EnergyPlus. The proposed technique was compared with the conventional air conditioning approach, which results in reduction in cost and energy use. In order to balance the occupant comfort and residential energy consumption, a technique based on ZigBee-based sensor nodes and a machine learning approach has been proposed in [33].

The impact of different dynamic prices like ToU and Critical Peak Pricing (CPP) has been detailed in a survey [34]. Here, 15 homes were taken for experiments where the presence of central air conditioning and Programmable Communicating Thermostat (PCT) have been ensured. Similarly, another survey was performed in order to check the use of thermostat in ambient temperature variations and quantitative interviews of users were conducted for the comfort evaluation in Finland [35]. Additionally, the effect of occupancy using occupancy-responsive thermostat was observed in [36]. When the building was vacant, an occupancy-responsive thermostat change the initialized set-point to the setback.

The Hidden Markov Model has been used to estimate the user occupancy status in [37]. A smart thermostat was proposed for turning the HVAC ON and OFF by following the user occupancy and sleep pattern. Motion sensors and door sensors were used to collect the data about a user schedule as well as simulations were performed in EnergyPlus. The proposed technique achieved better energy efficiency while the user comfort was heavily sacrificed because of the missing time, and the other limitation of this proposed technique is that it uses only one type of HVAC equipment for evaluating its proposed proposal. Information transmission in underwater wireless sensors is majorly disturbed by the energy holes. A localization-free interference and energy hole minimization routing protocol has been proposed in [38]. Simulation results of the proposed technique has shown the better performance in the number of packets received to the final destination. In order to increase the efficiency of demand response in HEMS, different types of research work are going on from a traditional method of control to a more efficient feedback controller. In [39], the occupancy of the user has been used to review Model Predictive Control (MPC) for HVAC buildings. A limitation of this approach is its high computational cost and complexity of designing a particular system.

A hybrid technique using two optimization approaches of Bacterial Foraging Algorithm (BFA) and genetic algorithm [40] with the objective of reducing cost and PAR has been proposed. PAR is an important performance metric used to evaluate the effect of peak electricity consumption on the system. There are a variety of appliances that require either high voltage or low voltage. Demand-side Management (DSM) persuades the electricity consumer to shift the high voltage required appliances to the low peak hours so that less energy is consumed during peak hours. However, most of the schedulers often result in the increase of PAR as they increase the load during low peak hours. The proposed algorithm used a fitness function of cost reduction, but it also minimizes PAR using day ahead RTP.

In [41], hybrid meta-heuristic techniques for scheduling of appliances have been proposed. The main point of designing a hybrid technique is a balance of exploration and exploitation. In order to achieve this balance hybrid of Optimal Stopping Rule (OSR) with Genetic Algorithm (GA), Teaching Learning Based Optimization (TLBO) and a Firefly Algorithm (FA) have been proposed. The objectives of this research were to reduce energy consumption and minimizing waiting time along with peak-to-average ratio and cost as optimized parameters. MATLAB (R2013a, MathWorks, Natick, MA, USA) simulation has been conducted while considering user priorities and RTP for heterogeneous homes. A limitation of the proposed methodology is the use of a limited number of appliances. This proposed scheme has not considered the effect of HVAC, which is the major energy consumer in the residential sector.

### 2.2. Fuzzy Inference System for DSM

A control strategy was developed for HVAC in order to respond to the RTP. A Dynamic Demand Response Controller (DDRC) [42] has been proposed that evaluates the value of electricity price at 15 min interval and change the thermostat set-point according to the setback initialized by the user. DDRC was implemented in MATLAB and the house model was integrated using EnergyPlus. A limitation of the proposed methodology is the use of a narrow range for temperature maintenance as they have not considered user preference.

In [43], a fuzzy logic rule-based algorithm was developed along with wireless sensors’ integration. A wireless programmable thermostat has been simulated in MATLAB. The proposed system used outdoor temperature, load demand, electricity price and user occupancy parameters in real time to reduce the thermostat set-points for better energy utilization in heating/cooling systems. Performance metrics are demand response participation, energy consumption, and the occupants’ user comfort. The Center of Gravity (CoG) method is used for defuzzification, whereas membership functions of parameters are defined as a triangular shape. As the output parameter decides how much load reduction should be done, the defuzzified value tells by how much value an initialized set-point will change. The objective of this research was to reduce energy consumption while having a best indoor temperature, but user comfort is sacrificed.

In the literature, it is found that a user often neglects or forgets to change the set-points according to varying price rates. In [44], an autonomous thermostat was developed by integrating fuzzy logic, wireless sensors, and smart grid initiatives. The proposed approach used Supervised Fuzzy Logic Learning (SFLL) including parameters of outdoor temperature, occupant presence, current electricity price and electricity demand to reduce the thermostat set-point. The proposed autonomous thermostat worked in two modes: (i) Economy Mode and (ii) Comfort Mode. The developed thermostat can handle all types of electricity pricing rates using Mamdani FIS. A limitation of the proposed system is its region-specific approach as research was conducted in a cold country—Canada.

Because of advancement in technologies and communication, the Programmable Communicating Thermostat (PCT) is often recommended for use. PCTs automatically change the set-point of a thermostat according to the change in the pricing rate. PCTs are the thermostat that does not require the constant user interaction. Comparison between the conventional thermostat and programmable communication thermostat has been performed in [45] using computer simulation that was run for 24 h. In this paper, price-based demand response program of RTP for residential air conditioning load is used to reduce the peak electric load. The authors have considered three air conditioners with different power ratings to perform simulation in SIMULINK (R2013a, MathWorks, Natick, MA, USA). In [46], Fuzzy Logic Approach (FLA) is augmented with PCT to increase the working efficiency of thermostat for load reduction that can work for both RTP and ToU pricing mechanisms. Demand response has been implemented as proactive demand shedding using MATLAB GUI. A house simulator using thermodynamic principles was described in this paper. The input parameters were current outdoor temperature taken from sensors; electricity price communicated by the utility; occupancy from occupancy sensor and initialized set-points of thermostat, whereas the output of the system is load reduction. The proposed technique (PCT + FLA) has been compared with fixed thermostat, programmable thermostat, and PCT, showing that proposed work outperforms in energy consumption minimization. A limitation of this technique is its non-adaptiveness.

The system proposed in [46] was made adaptive using Adaptive Fuzzy Logic Model (AFLM) in [17]. In this study, AFLM has been integrated with the wireless sensor network to learn and adapt the thermostat set-point according to the user priorities. Learning vectors and adapting vector were used in order to learn the change in user preference and adapt according to the user’s choice if the initialized set-points have been overridden by the user for consecutive three times. Simulations were performed for two months using “Manual Mode” and “Autonomous Mode” where “Autonomous Mode” was preferred by the user.

In [28], a worldwide adaptive thermostat controller by implementing fuzzy logic has been presented. The proposed controller changes the thermostat set-points as the load demand changes. Input parameters to this system were user occupancy, utility price, outdoor temperature, and initialized set-point. This system was evaluated using Mamdani FIS and Sugeno FIS. Performance metrics were energy consumption, cost, PAR, and user comfort. Energy consumption was computed using FIS by performing MATLAB simulations where Sugeno FIS outperformed among all the approaches. The proposed technique resulted in low cost and avoidance of peak formation, but it also results in user discomfort.

In [47], two demand-side management techniques i.e., load shifting and load curtailment, have been applied. In this research, the proposed techniques were Binary Particle Swarm Optimization Fuzzy Mamdani (BPSOFMAM) inference system and Binary Particle Swarm Optimization Fuzzy Sugeno (BPSOFSUG) inference system for controlling and scheduling electric loads. The proposed techniques were implemented on 10 single family apartments in order to control daily used appliances e.g., washer, dryer, etc. and seasonally used appliances e.g., air conditioner. BPSO was used to schedule daily used appliances during low peak hours, whereas fuzzy logic was used for maintaining thermostat set-points to improve the energy utilization efficiency. Air conditioning system set-points were initialized according to the PMV indexing method. Although the proposed technique outperforms in energy consumption minimization as compared to the existing approaches, user comfort is sacrificed. In literature, a fuzzy controller that aims to use ventilation for passive cooling of the residential building has been proposed in [48]. Cooling demand was reduced by optimizing the proposed controller using a multi-objective evolutionary algorithm. Although the proposed methodology considered the thermal comfort, the major focus was mainly on the cooling load of HVAC. A brief summary of the most relevant papers is presented in Table 2 only for quick lookup.

## 3. Problem Formulation

Fuzziness is a term that arises when we are dealing with uncertainty coming from linguistic concepts without a clear boundary. Measuring user comfort in HEMS is considered as vague or fuzzy. In natural language, for example, if someone classifies the user thermal comfort as cold, it leaves us with the uncertainty of how cold it is. Introduction of a fuzzy logic in the system will make the user interaction more natural [49]. Fuzzy logic allows researchers to compute with words that help in human reasoning and decision-making.

### 3.1. Fuzzy Logic Controller

**Figure 1:**
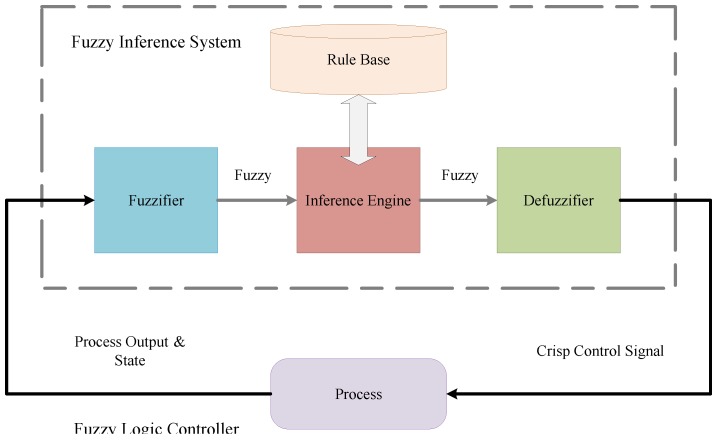
Conceptual diagram of the fuzzy logic controller.

Conventional controllers are expressed in the form of mathematical modeling so that they can represent the physical quantities of the real system. Inputs and outputs of Fuzzy Logic Controllers (FLC) are real variables that are mapped with a nonlinear function. Because of this, they have been found to be very helpful in modeling the nonlinear HVAC system. FLC is advantageous because of having no mathematical modeling requirement as compared to the conventional controllers. Conceptual diagram of the FLC system is shown in Figure 1.

FIS takes the crisp inputs, fuzzifies them, applies fuzzy operators on the premise (antecedent), performs implications from the premise to the conclusion (consequent), aggregates the conclusion across fuzzy rules to generate fuzzy output and defuzzify it to get a crisp output. The model proposed in this paper is evaluated and tested using the Mamdani FIS and Sugeno FIS. Mamdani FIS rules are generated using the linguistic variables for both the premise and conclusion.

IF Outdoor-Temp is “Normal” AND Indoor-Temp is “Normal” AND Rates is “High Peak” AND Occupancy is “Absent” AND ISP is “Low” AND Humidity is “Low” THEN energy consumption is “Low”.

The formula used for the centroid defuzzification method in Mamdani FIS is as follows [50]:

(1) $$z=\frac{\int \mu_{C}\left(z\right)\cdot zdz}{\int \mu_{C}\left(z\right)dz}.$$

Sugeno FIS takes the premise part as a linguistic variable; however, its conclusion part is a function that can be of zero order (constant) or first order.

IF Outdoor-Temp is “Normal” AND Indoor-Temp is “Normal” AND Rates is “High Peak” AND Occupancy is “Absent” AND ISP is “Low” AND Humidity is “Low” THEN energy consumption = energy-consumption ( $temp_{in}$, $temp_{out}$, price, occupancy, ISP, humidity).

The formula used by Sugeno FIS for the average weighted defuzzification method is as follows:

(2) $$z=\frac{\sum \mu_{C}\bar{z}\cdot \bar{z}}{\sum \mu_{C}\bar{z}}.$$

To conclude, Mamdani FIS is intuition based method that is well suited for the human input, whereas Sugeno FIS is a computationally efficient method and is well suited for the mathematical analysis [51]. In order to calculate the total cost of electricity consumption in this paper, the following formula is used where Cost(h) is the hourly cost, EC(h) is the electricity consumption on an hourly basis and Rates(h) are the hourly pricing tariffs based on ToU:

(3) Cost(h)=EC(h)×Rates(h).

PAR is used to get the information of the load peaks and to determine how much load balance is achieved. PAR is calculated using the formula below:

(4) $$PAR=\frac{Load_{maximum}}{Load_{average}}.$$

The efficiency of the proposed technique as compared to the previous technique is calculated from the equation below where $Value_{pre}$ is the value achieved using a previous approach and $Value_{pro}$ is the value obtained by applying the proposed technique:

(5) $$Efficiency\;Gain=\frac{Value_{pre}-Value_{pro}}{Value_{pre}}.$$

### 3.2. The Proposed Model

There are many parameters that can be considered as an input to the rule base of the fuzzy logic system. However, proposed research considers parameters that directly influence the user comfort and energy consumption minimization. This research investigates the inclusion of humidity as an input parameter to proposed FIS and adding feedback loop to the system in order to maintain the user comfort and reduce the energy consumption. The parameters used in the FIS are indoor temperature, outdoor temperature, pricing mechanism, occupant’s presence, initialized set-point and relative humidity of the current house or region. In order to measure the real-time environmental variables, the use of wireless sensors is required. The proposed controller in this paper has the potential to be embedded in the IoT operating system RIOT as IoT increases the number of parameters to be monitored along with increase in the efficiency of control over the internet. FIS is used to decide how much energy consumption should be made by the controller.

**Figure 2:**
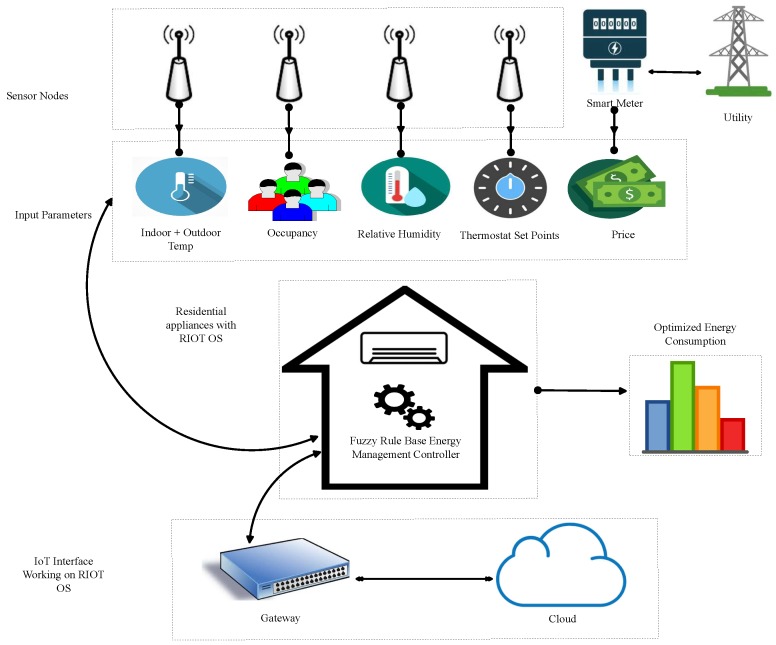
Block diagram of the proposed fuzzy inference system.

Figure 2 shows that environmental variables of the surroundings are monitored by the sensors. Crisp values of the input parameters i.e., indoor temperature, outdoor temperature, humidity, user occupancy and the initialized set-points are continuously sent to controllers and the measurements are periodically sent to the Internet over the gateway. It is assumed that the proposed technique communicates with a smart meter in order to read the price signal imposed by the utility. Initialized set-points on the thermostat are decided by the user on the basis of environmental conditions i.e., humidity and temperature. Energy management controller utilizes the fuzzy rule base inference system to make the decision regarding energy consumption based on the combinations of sensor values and smart grid price values by firing the corresponding rules. Decision on energy consumption is sent to HVAC by an actuator for the desired working of appliances.

In addition, indoor temperature change is also monitored at 5 min intervals and sent to the controller through a feedback loop for the heater room model. After every 5 min, it is decided whether the heater should be ON or OFF on the basis of indoor temperature and thermostat set-point. A number of simulations have been performed to decide the optimal time interval for measuring the room temperature variation. If the interval is less than 5 min, a heater continuously switches between ON and OFF state, which is not suitable for energy conservation. However, if the interval is set for more than 5 min, simulation results have shown that the indoor temperature rises above the thermostat set-point that can affect the user thermal comfort. Because of this, a 5 min interval is considered as the optimal interval that maintains the balance between energy conservation and user comfort. In order to switch the heater ON or OFF, a relay is used. When the temperature of the room changes in accordance with the thermostat set-point proposed controller will send the command to an actuator that controls the working of the heater. Our fuzzy inference rule base takes 972 bytes of memory and our entire proposed system will have memory requirements of 2 kB. Less memory requirements and low processing power to traverse lookup tables make it ideal to be used with any IoT operating system. Operating system used for IoT software is RIOT. RIOT is a microkernel-based operating system that matches with various software requirements of devices and deals with challenges related to the constraint of memory and power consumption. RIOT has the ability to work for small networked devices that provide real-time controlling capabilities with less memory constraints. All of these features of RIOT make it a perfect choice to implement the proposed controller on system. In order to generate the fuzzy rule base system using the input parameters, a combinatorics method is devised that defines a total of 486 rules automatically, which is otherwise difficult to program manually.

As a result, the expectations of a “Controller” that is equipped with proposed FIS are as follows:

(i) A system that helps the user to set a wide range of the thermostat set-points based on the temperature and relative humidity.
(ii) An energy management controller that will help in better energy optimization.
(iii) A controller that can handle smart grid initiatives like ToU and DR that results in the energy management and conservation.
(iv) The proposed controller will help in initializing the thermostat set-points that maintain the temperature under user comfort zone.
(v) FIS controller is designed in such a way that can include other parameters without having the hassle to define a large number of rules.
(vi) The proposed controller includes room temperature variation that helps in making better decisions for the energy conservation.
(vii) Using an IoT based fuzzy controller will result in real-time monitoring and total controllability.

A thermodynamic model is used to measure the room temperature variation in order to simulate the indoor temperature sensor. The input parameters of the proposed feedback loop system are indoor temperature, outdoor temperature, initialized set-point, occupancy and the electricity price. In [52], the authors have discussed the difficulty and complexity of modeling room temperature variation and at the same time catering to the effect of external weather so that outside climate varies continuously. The principle of heat transfer [53] is developed from thermodynamic laws and rate equations of conduction, convection, and radiations. The proposed research only considers the conduction and convection mechanism [54,55] to represent the thermodynamic model of the room. The thermodynamic model used is verified and analyzed with respect to different energy managing parameters.

### 3.3. Model of Residential Heating System

In order to model the working of the heater inside a room and simulate the variation in room temperature, heater and its thermal characteristics, room and its thermal characteristics and a thermostat are taken as a model component. The thermal model is an adaption of the [46], which was created using the model of heat gain and heat loss of system.

**Figure 3:**
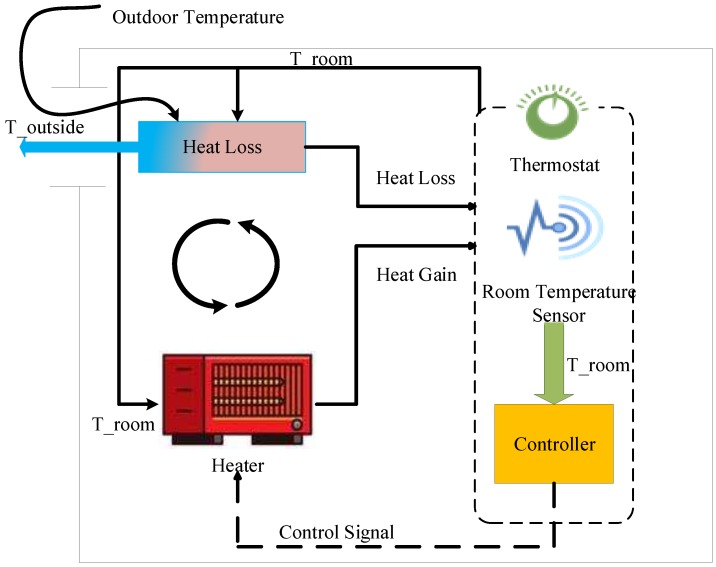
Heater model with thermostat controller.

As shown in Figure 3, thermostat plays a central role in the room-heater model with having a room temperature sensor and controller. In this model, heat generated by the heater is calculated as well as the heat lost to the surroundings. After all the calculations have been performed, the controller part of the thermostat reads the value of the indoor temperature and sends the control signal if the room temperature is different as compared to the set-point temperature. The components of residential heating system are described below:

1. Heater:
  Heater component is modeled using the amount of heat gain supplied to the room. When the heater is ON, hot air is blown at a constant temperature $T_{Heater}$ and a flow rate $M_{Heater}$. Heat gain inside the room is calculated using the equation below. Output of the heat is dependent on the thermostat control signal:
  (6) $$\frac{dQ_{gain}}{dt}=\left(T_{Heater}-T_{Room}\right)\times M_{Heater}\times c_{air},$$
  where $\frac{dQ_{gain}}{dt}$ is heat gain because of hot air flow from heater, $T_{Heater}$ is temperature of hot air from heater, $T_{Room}$ is room air temperature at real time, $M_{Heater}$ is air mass flow rate through heater, and $c_{air}$ is heat capacity of air at constant pressure.
2. Thermostat:
  Thermostat in the room calculates the difference in the initialized thermostat value and room temperature and turns the heater ON or OFF based on the difference. Thermostat computes the difference at each 5-min interval. The working of the thermostat can be summarized as follows:
  (i) When the room temperature is below the desired set-point, the heater state is ON and it supplies the heat gain and value of control signal is equal to 1.
  (ii) When the room temperature is above the initialized set-point for that particular time, the heater is turned OFF by the thermostat and heat gain is equal to zero as the control signal is 0.
3. Room:
  In order to calculate the temperature variation, the system considers both the heat gain from the heater and heat loss from room to the surroundings. Heat loss ($\frac{dQ_{loss}}{dt}$) was calculated using the equation mentioned below where $T_{outside}$ is outdoor temperature at real time and $R_{thermal}$ is equivalent thermal resistance of the house:
  (7) $$\frac{dQ_{loss}}{dt}=\frac{T_{Room}-T_{outside}}{R_{thermal}}.$$
  In order to compute the variation in indoor temperature ($\frac{dT_{room}}{dt}$), heat gain and heat loss calculated using the Equations (6)–(7) two formulas were added in the following equation, where $M_{air}$ is the mass of air inside room:
  (8) $$\frac{dT_{room}}{dt}=\frac{1}{M_{air}\times c_{air}}\left(\frac{dQ_{gain}}{dt}-\frac{dQ_{loss}}{dt}\right).$$

In order to validate the proposed methodology, two extremal temperatures are considered i.e., coldest and hottest day from Yakutsk, Russia.

## 4. System Model Implementation

In this proposed technique, parameters have been considered that directly affect the energy consumption and user comfort in residential buildings. These parameters are indoor temperature ($Temp_{indoor}$), outdoor temperature ($Temp_{outdoor}$), user occupancy (Occ), price rates ($P_{rates}$), thermostat set-points ($ISP_{s}$), and humidity ($Humidity_{rel}$).

In the proposed technique, trapezoidal membership function is used to define the membership functions of the input and output variables. This system has only one output that is energy consumption (EC). Although the triangular membership function is simple to use, the parameters used in this study are best defined using trapezoidal membership function since the temperature, set-points, price and humidity do not suddenly drop their value and maintain the same value for a length of time. Thus, these flat line membership functions have the advantage of simplicity [50].

### 4.1. Outdoor Temperature

Electricity load is affected by different variables like outdoor temperature and electricity prices. There is a strong relationship between setting the set-point and outdoor temperature that directly determine the energy consumption. The importance of outdoor temperature becomes more valuable when the temperature fluctuates strongly in many countries like Canada. This situation results in making the HVAC a highly variable load among all the appliances. Generally, when the outdoor temperature is very cold or hot, the electricity demand is very high. As this system is an extension of an adaptive fuzzy learning model [17] and a worldwide adaptive thermostat model [28], two extreme weather details have been considered.

**Figure 4:**
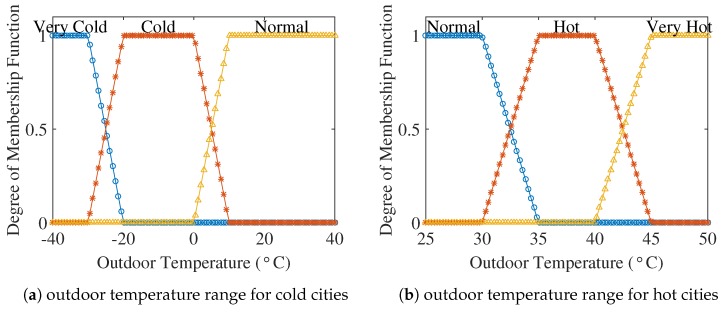
Membership functions of outdoor temperature.

Two cities considered for analyzing the cooling and heating power consumption in any residential building are the Wadi Halfa in Sudan and Yakutsk from Russia. Wadi Halfa is one of the hottest cities in the world and Yakutsk is the coldest city in the world. After that, one of the hottest and coldest days from the respective cities is taken into account. Wadi Halfa experiences the highest temperature during the month of June, whereas Yakutsk is coldest during January. The outdoor temperature for Wadi Halfa and Yakutsk was taken from a weather forecasting website [56,57], respectively. The outdoor temperature is considered on an hourly basis. Figure 4 shows the membership functions of the outdoor temperature.

### 4.2. Indoor Temperature

**Figure 5:**
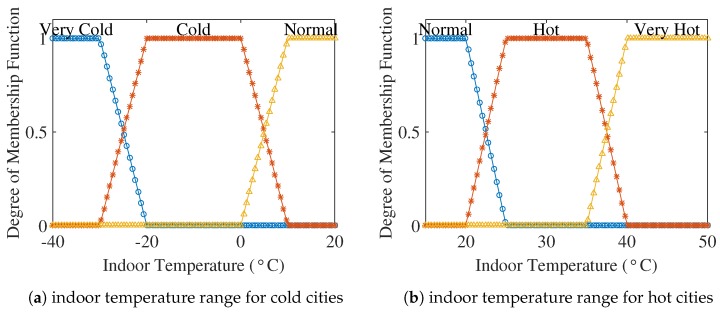
Membership functions of indoor temperature.

Indoor temperature is an important parameter that determines the user comfort. Energy consumption is highly dependent on the indoor temperature. If the outdoor temperature is at the extreme, then maintaining a normal indoor temperature often results in high energy consumption. Figure 5 defines the membership function of the indoor temperature used for hot and cold cities.

### 4.3. Occupancy

**Figure 6:**
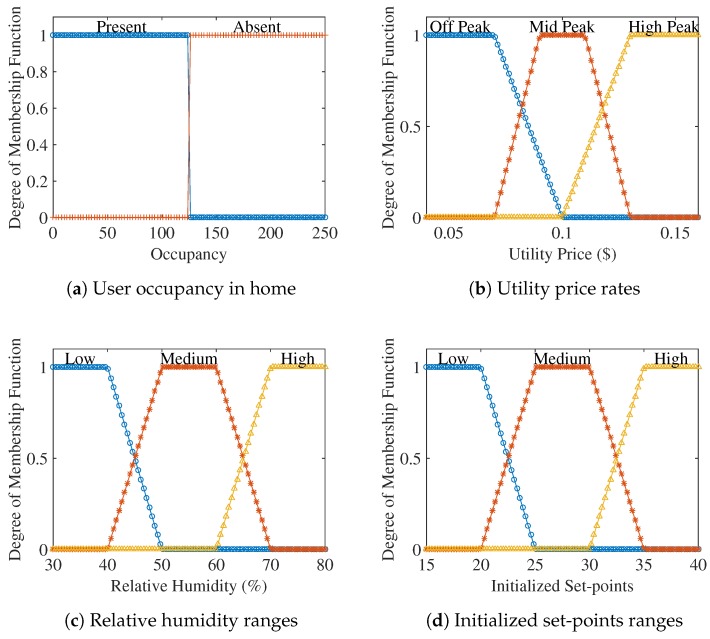
Input membership functions of occupancy, price, relative humidity, initialized setpoints.

Occupancy is a parameter that determines the importance of user comfort. Occupancy data to know whether a person was present at a particular time or not can be received from occupancy sensors. The effect of occupancy can be seen in the energy consumption because, when a place is not occupied, a proposed controller will work differently as compared to a scenario when that place is occupied. Hence, considering the effect of user occupancy not only results in energy consumption minimization, but it also maintains user comfort. Membership function for occupancy input parameter is shown in Figure 6.

### 4.4. Price Tariff

**Figure 7:**
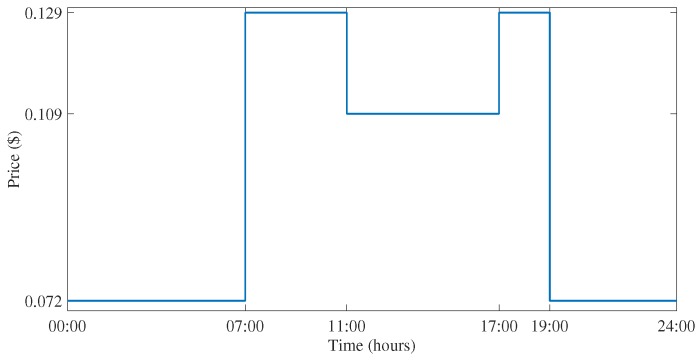
Time of Use rates by Hydro One, Ontario, Canada.

Reduction in bill is a target that residential users always want to achieve. Total bill and energy consumption shows high dependence on the pricing tariffs for the current time. In this proposed methodology, a ToU pricing tariff is considered. Installing smart meters is very advantageous for residential users as they provide consumers with current energy cost as well as current energy consumption. This kind of interaction will reduce the peak load as the user can reduce the load during peak hours, which will benefit both utility and user by decreasing the energy consumption and resulting in bill reduction. Figure 7 shows the price tariff from Hydro One, Canada used for the evaluation of results in this paper.

### 4.5. Relative Humidity

**Figure 8:**
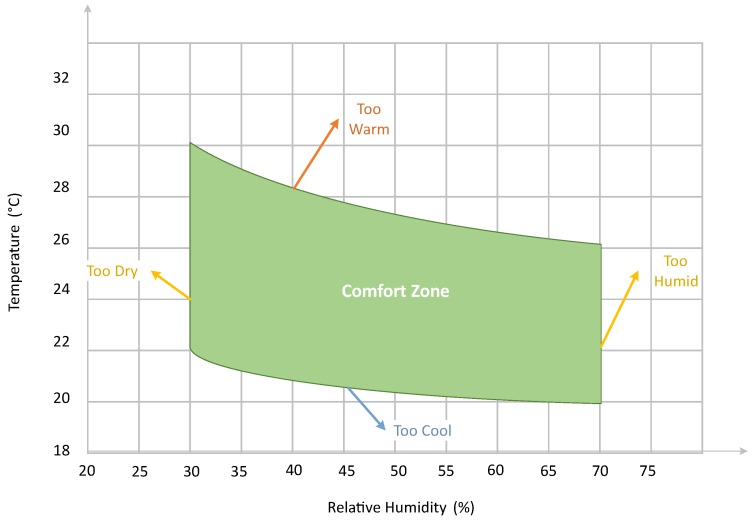
Depiction of user comfort zone.

One of the parameters that has a huge impact on the evaluation of thermal comfort is humidity. Integrating the values of relative humidity not only helps to reduce the energy consumption but also maintains the environment that lies in the user comfort zone. There is a range of temperature and humidity values that lies in the user comfort zone according to the American Society of Heating, Refrigerating and Air-Conditioning Engineers (ASHRAE). It can be seen in Figure 8 that a user can be in a comfort zone even at a higher temperature if the humidity is low [58]. However, if temperature reduces, higher humidity maintains the user comfort zone.

### 4.6. Initialized Set-Points

The proposed solution simulated the working of the thermostat. The user sets the thermostat set-points according to their need and comfort. Therefore, initialized set-points are considered as one of the inputs to the FIS in order to measure the energy consumption. The proposed system monitored the initialized set-points on an hourly basis and, along with the other information, helped in applying the appropriate rule. Initialized set-points defined for indoor temperature for both hot and cold cities were set according to the psychrometric chart mentioned in [58].

### 4.7. Automatic FIS Rule Base Generation

The proposed system is evaluated with the help of fuzzy rules in order to determine the total energy consumption. As the input parameters of the FIS increase, the number of rules in the FIS rule base also increased. It is often observed that defining rules manually to the software is a very lengthy and tedious process. As the proposed technique in this paper is an extension of a worldwide adaptive thermostat model [28] by adding humidity as an input parameter, one can observe the increase in the number of rules, which is as follows:

(i) In worldwide adaptive thermostat [28], there were four variables with three membership functions and one variable with two membership functions. This resulted in a total of 162 rules to be defined in the rule base of Mamdani FIS and Sugeno FIS.
(ii) Adding humidity as a parameter to the system results in five variables with three membership functions and one variable with two membership functions. A total of 486 rules are required to be defined for the both Mamdani FIS and Sugeno FIS.

As the number of rules to be defined in a system increases, the process of defining the rules will become cumbersome. Many approaches have been designed to lessen the burden of defining rules manually. In this paper, a technique for the automatic generation of rule base is proposed using the combinatorics and weightage method. Each membership function of a single input parameter is assigned a weightage that is used to calculate Score, and values of output parameters are going to be assigned according to the calculated Score as shown in Algorithm 1. The major steps of FLC are as follows:

(i) The first step is the fuzzification process in which all the membership functions of the system parameters are initialized and defined.
(ii) The second step is defining the rules in the rule base by giving weightage to membership functions of input parameters and then assigning the suitable output fuzzy value.
(iii) The third step uses the Mamdani FIS and Sugeno FIS to evaluate the energy consumption.
(iv) After rule evaluation, defuzzification is performed to get the crisp value for the energy consumption. In the end, calculation of remaining performance measures is performed.

In the second step, after assigning weights to the membership functions of the input parameters, to circumvent the combinatorial growth of IF-THEN rules, we introduce a parameter called Score, which is indicative of energy consumption. Low Score means low energy consumption and vice versa. The Score is calculated as the weighted sum of weights assigned to the membership function of variables involved. The weights are dependent on the membership function where membership function of low, medium, and high have the weights of 0, 1, and 2, respectively.

The only exception is the variable of Occupancy with binary weights of 0 or 1 in reference to the absence and presence of occupants. The method used for the automatic rule generation utilizes the combination of the input parameters where order does not matter and they follow the commutative property when Score is calculated by adding the weights of membership functions of input parameters. The Score at any instant is as follows:

(9) $$\begin{array}{cc} Score & =\sum_{i=1}^{6}W\left(v_{i}\right), \end{array}$$

where *W* is weight and variables $v_{i}$ are renamed according to Table 3.
**Algorithm 1** Automatic Rule Generator1:$Temp_{outdoor}\leftarrow$ {L,M,H}2:$Temp_{indoor}\leftarrow$ {L,M,H}3:$Humidity_{rel}\leftarrow$ {L,M,H}4:$P_{rates}\leftarrow$ {OP,MP,HP}5:Occ← {A,P}6:$ISP_{s}\leftarrow$ {L,M,H}7:**for** $Humidity_{rel}\left[1\right]$ to $Humidity_{rel}\left[n\right]$ **do**8:    **for** $Temp_{outdoor}\left[1\right]$ to $Temp_{outdoor}\left[n\right]$ **do**9:        **for** $Temp_{indoor}\left[1\right]$ to $Temp_{indoor}\left[n\right]$ **do**10:           **for** $P_{rates}\left[1\right]$ to $P_{rates}\left[n\right]$ **do**11:               **for** $ISP_{s}\left[1\right]$ to $ISP_{s}\left[n\right]$ **do**12:                   **for** Occ[1] to Occ[n] **do**13:                       Compute Score          ▹ Defined in Equation (9)14:                       **if** Score = 0 or Score = 1 **then**15:                          EC = VL16:                       **else if** Score = 2 or Score = 3 **then**17:                          EC = *L*18:                       **else if** Score >= 4 and Score <= 7 **then**19:                          EC = *M*20:                       **else if** Score = 8 or Score = 9 **then**21:                          EC = *H*22:                       **else**23:                          EC = VH24:                       **end if**25:                   **end for**26:               **end for**27:           **end for**28:        **end for**29:    **end for**30:**end for**


Sample FIS rules defined using the automatic rule generation algorithm are presented in Table 4. Membership functions of the input provided to the proposed FIS and the resulting output are defined and abbreviated as follows:

(i) Temperature: Indoor temperature ($T_{in}$) and outdoor temperature ($T_{out}$) for both hot and cold cities have membership functions of (1) Low (L); (2) Medium (M); and (3) High (H) according to their cold and hot weather.
(ii) Electricity Pricing: Pricing tariff (Rate) is classified into the following membership functions: (1) Off-Peak (OP); (2) Mid-Peak (MP); and (3) High-Peak (HP).
(iii) Occupancy: The user can either be present in the residential building or not. This condition is demonstrated using the membership functions of (1) Absent (A) and Present (P).
(iv) Thermostat set-points and Humidity: Both initialized set-points (ISP) and humidity (Humidity) input parameters are classified in the membership function of (1) Low (L); (2) Medium (M); and (3) High (H).
(v) Energy consumption: Output of the proposed FIS is energy consumed (EC), which is classified into the following membership functions: (1) Very Low (VL); (2) Low (L); (3) Medium (M); (4) High (H); and (5) Very High (VH).

## 5. Simulation Results

This section contains the simulation analysis of the proposed schemes. In order to investigate the effect of adding feedback loop in the FIS, a 2 kW heater is considered for a small room, whereas impact of adding a humidity parameter is observed using the 10 kW HVAC in a residential building. The objective of enhancing user comfort was achieved by initializing the set-points according to the temperature and relative humidity. The effect of adding a feedback loop in the FIS for energy consumption minimization is also discussed in this section.

### 5.1. Results of FIS with Feedback

**Figure 9:**
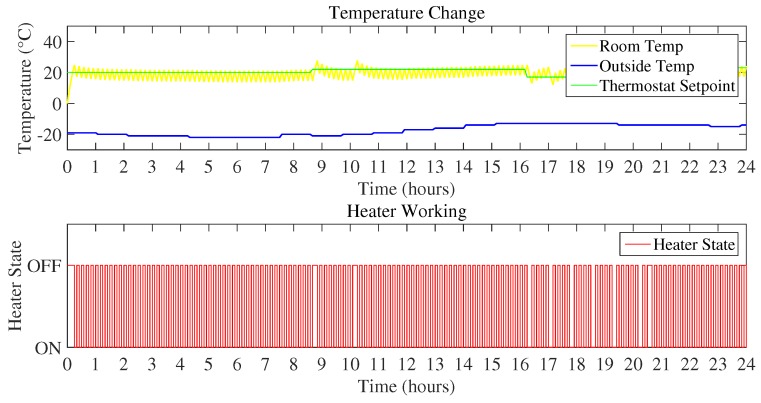
Temperature variation and heater state (Scenario I).

1 Scenario I:
  In Scenario I, outdoor temperature represented the coldest day that is below 0 ${}^{\circ }$C. As the temperature is very low, it is likely to take more energy consumption to maintain the inside temperature according to the user desired thermostat set-point. Figure 9 represents the room temperature variation with respect to the initialized set-points, outdoor temperature, and the heater state.

**Figure 10:**
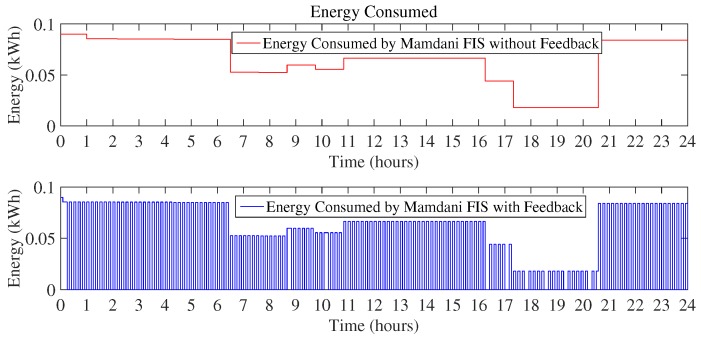
Energy consumption in a day using Mamdani FIS (Scenario I).

**Figure 11:**
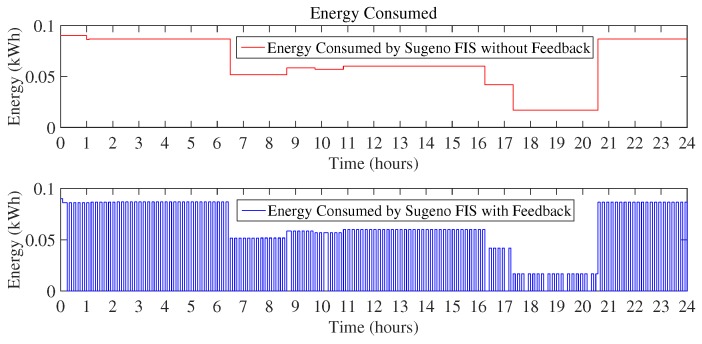
Energy consumption in a day using Sugeno FIS (Scenario I).

Figure 10 and Figure 11 show the daily energy consumption graph that is the output of using Mamdani FIS and Sugeno FIS with and without using a feedback loop. Figure 10 shows the comparison of Mamdani FIS when using feedback loop and without a feedback loop. As the outdoor temperature is very low, the heater is ON at a very large number of instances. It will constitute more energy consumption as compared to the other scenarios considered here. According to the Figure 10, energy consumption in a day using Mamdani FIS with feedback is 10.41 kWh and without feedback is 20.61 kWh.

As the proposed technique used two FIS, Figure 11 shows the energy consumption by using Sugeno FIS including feedback loop and without a feedback loop. Energy consumption using Sugeno FIS without a feedback loop is 20.38 kWh and FIS with the addition of the feedback loop consumes 10.30 kWh of energy.

**Figure 12:**
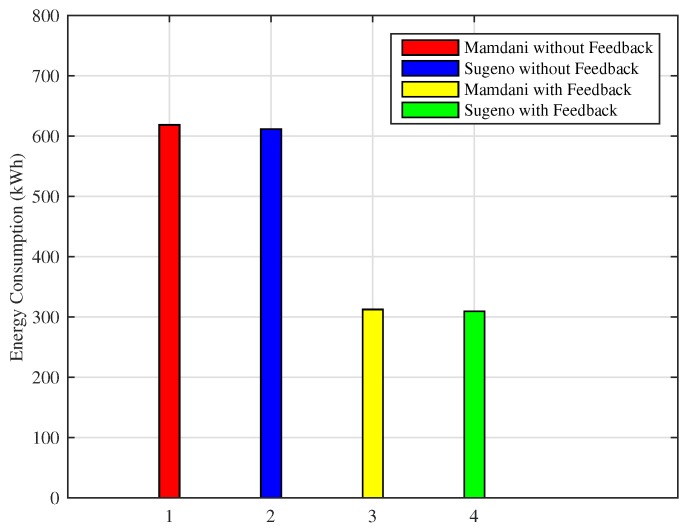
Monthly energy consumption (Scenario I).

Figure 12 shows the comparison of the proposed technique with the existing technique to show the effect of adding a feedback loop. As the energy consumption is computed at every 5 min interval by firing the rules from the fuzzy rule base that corresponds to the real-time scenario, it can be seen that fuzzy rule base with a feedback loop is getting realistic data, on the basis of which it turns the heater ON and OFF and saves the energy consumption accordingly.

**Figure 13:**
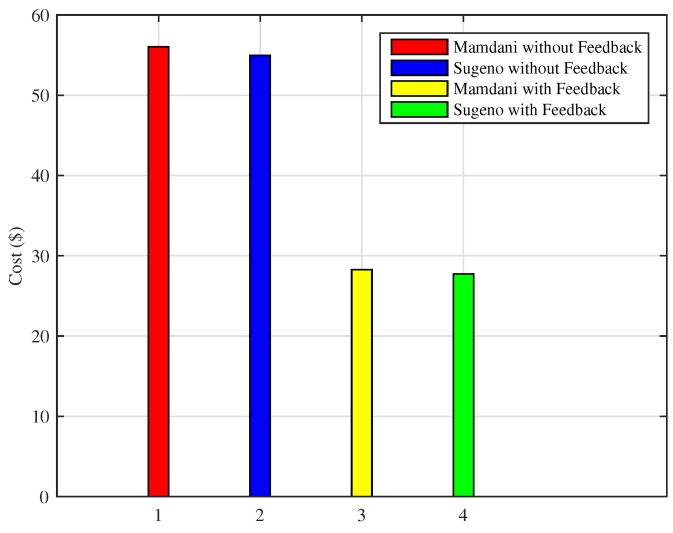
Monthly electricity cost incurred (Scenario I).

Monthly consumption of the proposed Mamdani FIS is 312 kWh as compared to the existing Mamdani FIS where total energy consumption in a month is 618 kWh. Energy consumption in a month using Sugeno FIS with a feedback loop in this scenario is 309 kWh and without including feedback loop results in the 611 kWh of monthly energy consumption. In this scenario, it can be clearly observed that Sugeno FIS outperforms in improving the efficiency of energy consumption minimization. The monthly cost incurred to the residential consumers using the proposed techniques versus existing technique is presented in Figure 13. According to the graph, the monthly cost for Mamdani FIS with a feedback loop is $28.26 and with Sugeno FIS along with the feedback loop is $27.73. Energy consumption cost in a month without adding a feedback loop and using Mamdani FIS and Sugeno FIS is $56.05 and $54.95, respectively. From the above discussion, it can be concluded that Sugeno FIS is clearly the best performer among other considered methods.

**Figure 14:**
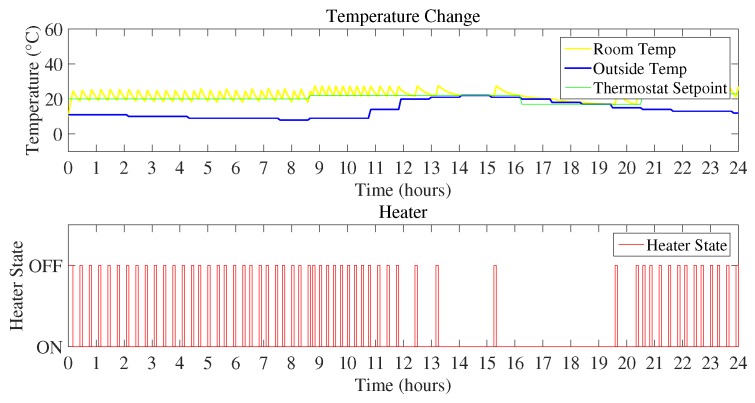
Temperature variation and heater state (Scenario II).

2 Scenario II:
  Outside temperature in this scenario depicted a sunny day where the outdoor temperature reached the desired thermostat temperature, in this case there was no need to turn ON the heater and the simulation results shown in Figure 14 depict the same behavior of the heater state in response to outdoor and indoor temperature. During the afternoon, outdoor temperature rises and the heater is kept in an OFF state in order to conserve the energy, as compared to the previous technique, where the heater is still ON and is utilizing the energy because the previous approach does not consider the variation in the room temperature.

**Figure 15:**
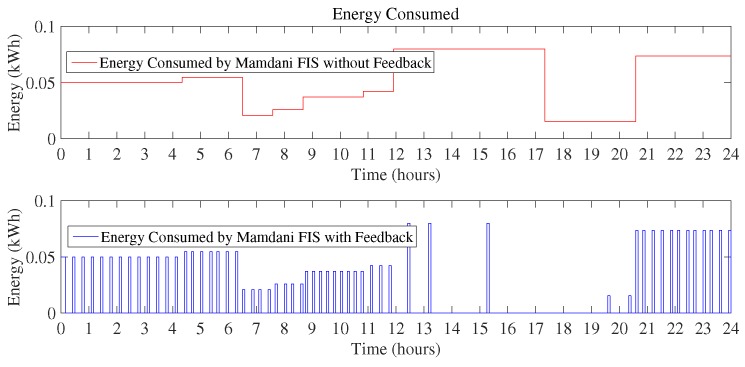
Energy consumption in a day using Mamdani FIS (Scenario II).

**Figure 16:**
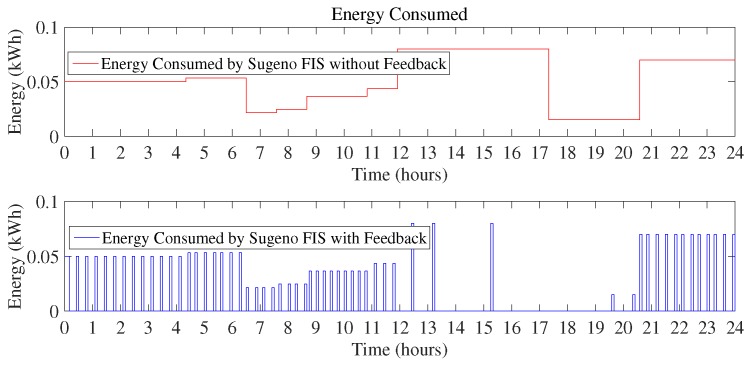
Energy consumption in a day using Sugeno FIS (Scenario II).

In this scenario, the outdoor temperature rises during the afternoon up to the desired thermostat set-point. During this time, there is no need for the heater to be turned ON and thus zero energy is consumed during these hours. Figure 15 and Figure 16 clearly validate the working of the proposed FIS as the energy is not being consumed during those hours and the energy is conserved. As compared to the existing technique of Mamdani FIS, which consumes 16.71 kWh in the day, Mamdani FIS with a feedback loop consumes 3.39 kWh. The minimum amount of energy used in this scenario by using Mamdani FIS without a feedback loop is 0.015 kWh, whereas, when feedback is added to the Mamdani FIS, it can reduce the energy consumption to 0 kWh. The maximum amount of energy consumed in both cases observed is the same, i.e., 0.08 kWh.

Similar behavior can be observed by looking at Figure 16. Energy consumed using Sugeno FIS without a feedback loop is 16.43 kWh, as compared to the total energy consumed in a day using Sugeno FIS with a feedback loop of 3.31 kWh, which increases the efficiency of the proposed FIS to achieve the objective of minimization of energy consumption.

**Figure 17:**
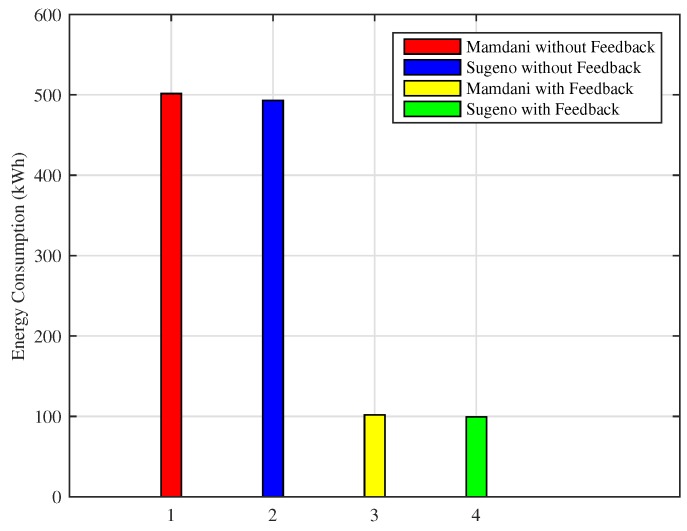
Monthly energy consumption (Scenario II).

Similarly, if the energy consumption in a month is observed in Figure 17, the proposed FIS outperforms as compared to the previous technique. Energy consumed in a month using Mamdani FIS and Sugeno FIS without adding a feedback loop is 501 kWh and 493 kWh, respectively, whereas the total amount of energy consumed during a month using Mamdani FIS and Sugeno FIS with the addition of feedback loop is 101 kWh and 99 kWh, respectively.

**Figure 18:**
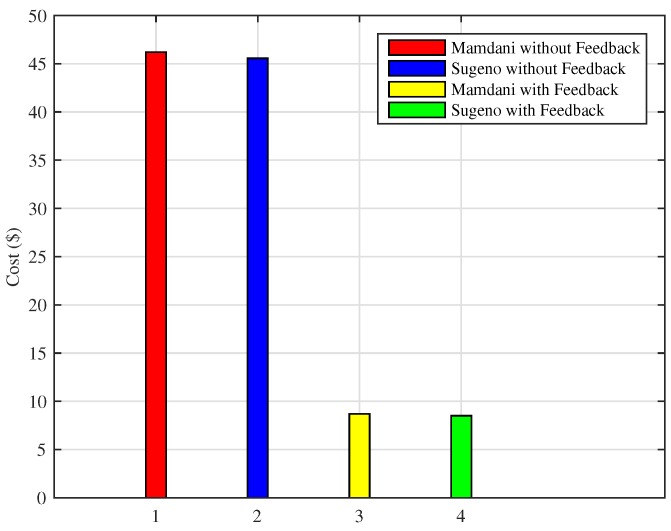
Monthly electricity cost incurred (Scenario II).

Consequently, the effect of conserving energy consumption can be seen in the total cost reduction. Figure 18 shows the total cost incurred by a residential building consumer using Mamdani FIS and Sugeno FIS with and without adding the feedback loop on the basis of the indoor room temperature variations. In the case of Mamdani FIS with and without the feedback loop, total cost of the energy used in a month is $8.68 and $46.19, respectively, whereas the cost while using Sugeno FIS with and without adding a feedback loop is $8.50 and $45.55, respectively. Similar to previous results, Sugeno FIS outperforms in terms of efficiency when energy and cost is considered.

### 5.2. Results of FIS with Humidity

1 Energy consumption with proposed FLC in Hot Cities

**Figure 19:**
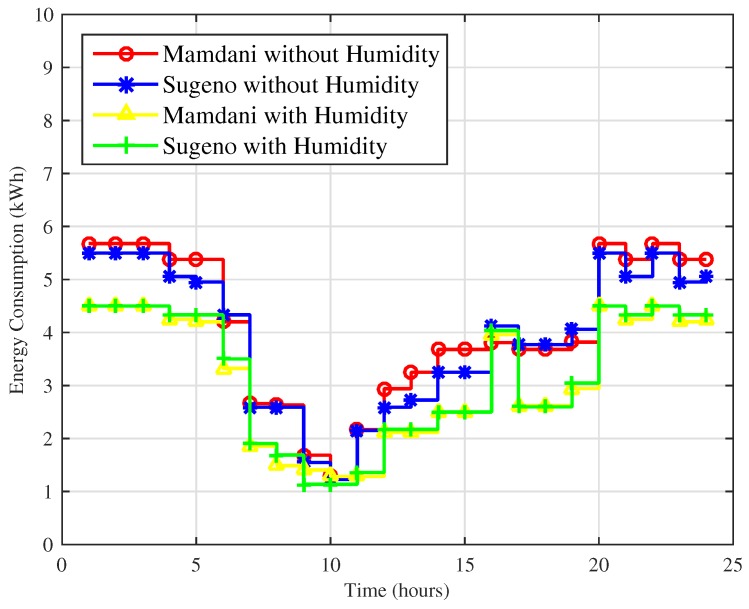
Energy consumption over a day for hot cities.

**Figure 20:**
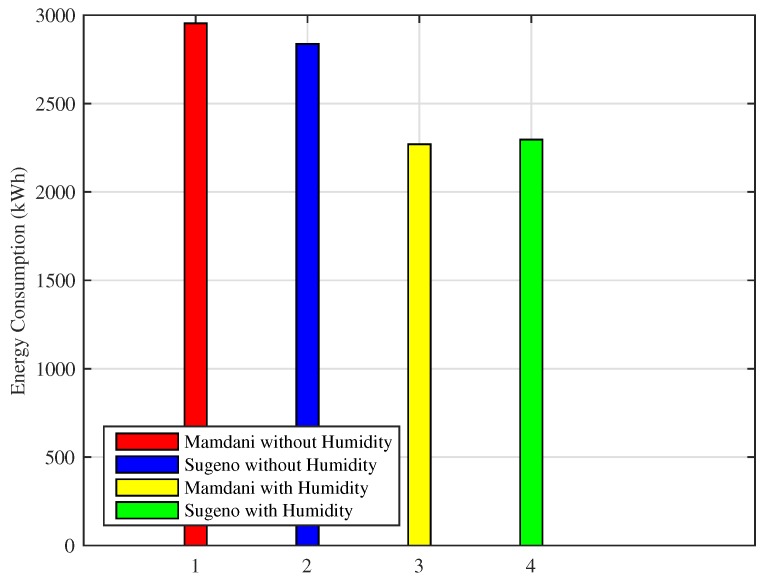
One month simulation of energy consumption for hot cities.

Two different simulation results for energy consumption in a day and for a month for comparison of techniques using and without using humidity as an input parameter are shown in Figure 19 and Figure 20.

In Figure 19, energy consumption for one day using Mamdani FIS and Sugeno FIS including and without including humidity is shown. Maximum hourly consumption for Mamdani FIS and Sugeno FIS without humidity is 5.7 kWh and 5.5 kWh, whereas maximum hourly energy consumption of proposed FLC using Mamdani FIS and Sugeno FIS with humidity is 4.5 kWh and 4.5 kWh. Our proposed FLC improves the energy consumption by effectively maintaining the user comfort up to 21% for both techniques.

Figure 20 shows the monthly energy consumption of our designed FLC using both FIS with and without considering humidity. Energy consumption shown is calculated by evaluating and analyzing the fuzzy rule base.

The energy consumption of FLC using Mamdani FIS without humidity is 2954 kWh, Sugeno FIS without humidity consumes 2837 kWh, Mamdani FIS with humidity shows 2270 kWh energy consumption and Sugeno FIS with humidity consumes 2295 kWh energy. Mamdani FIS with humidity improves energy consumption by 23% while Sugeno FIS with humidity is improving energy consumption by 22% as compared to the energy consumed using Mamdani FIS and Sugeno FIS without humidity.

The proposed Mamdani FIS performs better than proposed Sugeno FIS because it is simple in nature and has more energy efficiency. As the demand for HVAC varies on an hourly basis in a residential building, the set-points of the thermostat are modified by using temperature and relative humidity information. These thermostat points have been initialized keeping in mind the relationship of temperature and relative humidity according to the psychrometric graph so that user comfort is not jeopardized.

2 Energy consumption with proposed FLC in Cold Cities

As the proposed technique is the extension of [28], dealing with both hot and cold cities, this section discusses the energy consumption evaluation for the cold cities. Schedule of user occupancy and ToU rates remain the same for cold cities’ evaluation. Outdoor temperature, indoor temperature, relative humidity, and thermostat set-point were used in accordance with cold city weather data.

**Figure 21:**
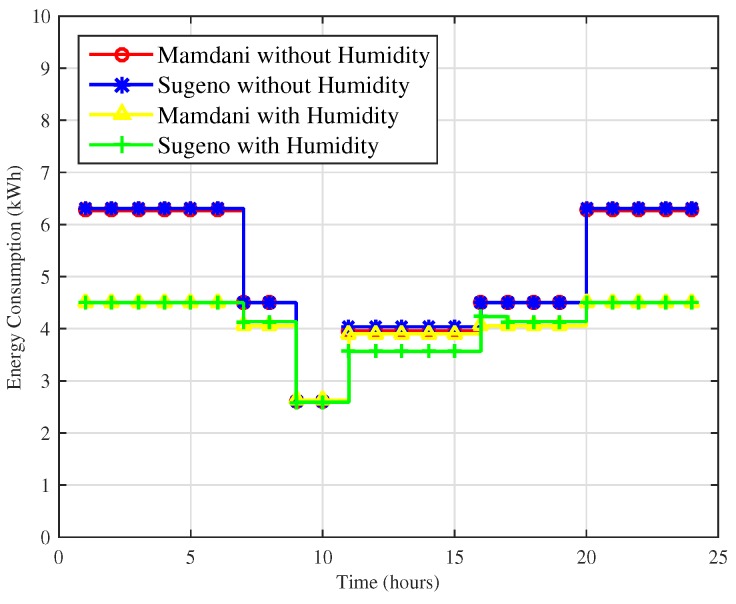
Energy consumption over a day for cold cities.

In Figure 21, energy consumption of the method proposed and the existing method for comparison is presented. The maximum energy consumption in cold cities is 6.26 kWh, 6.30 kWh, 4.5 kWh, and 4.5 kWh using FIS Mamdani FIS without humidity, Sugeno FIS without humidity, Mamdani FIS with humidity and Sugeno FIS with humidity. In a cold city scenario, Mamdani FIS and Sugeno FIS with humidity show 28% efficiency in energy consumption compared to FIS without humidity.

**Figure 22:**
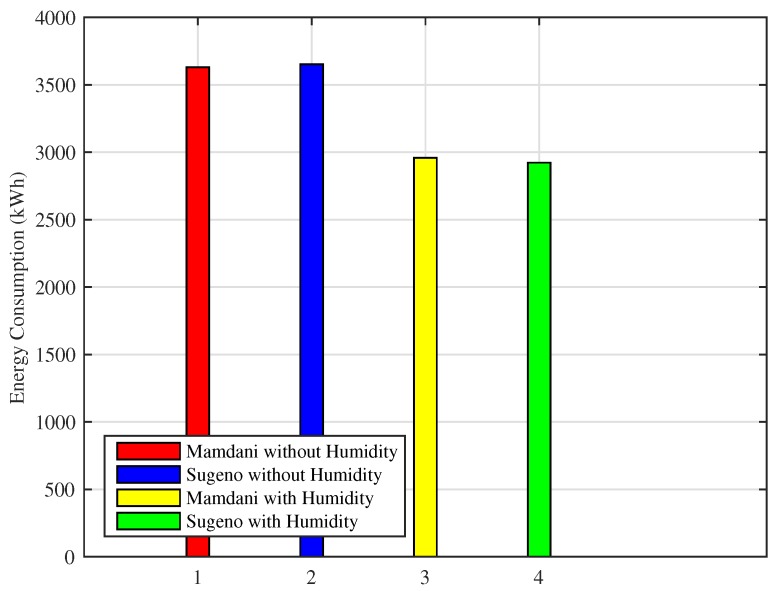
One month simulation of energy consumption for cold cities.

Figure 22 shows the energy consumption of cold cities for one month simulation using Mamdani FIS and Sugeno FIS while considering and leaving the humidity parameter. Energy consumption is minimized while maintaining user comfort at a desired level using $ISP_{s}$. Although the energy consumption for cold cities is greater as compared to hot cities, our proposed system succeeds in energy consumption minimization, which shows the efficiency of the proposed scheme compared to the earlier schemes.

Monthly energy consumption of Mamdani FIS without humidity is 3629 kWh, Sugeno FIS without humidity is 3652 kWh, Mamdani FIS with humidity uses 2958 kWh and Sugeno FIS with humidity consumes 2922 kWh of energy. Efficiency in energy consumption for Mamdani FIS with humidity is 19%, whereas Sugeno FIS with humidity is 20% as compared to both FIS without humidity.

3 Total Cost incurred with proposed FLC in Hot Cities

**Figure 23:**
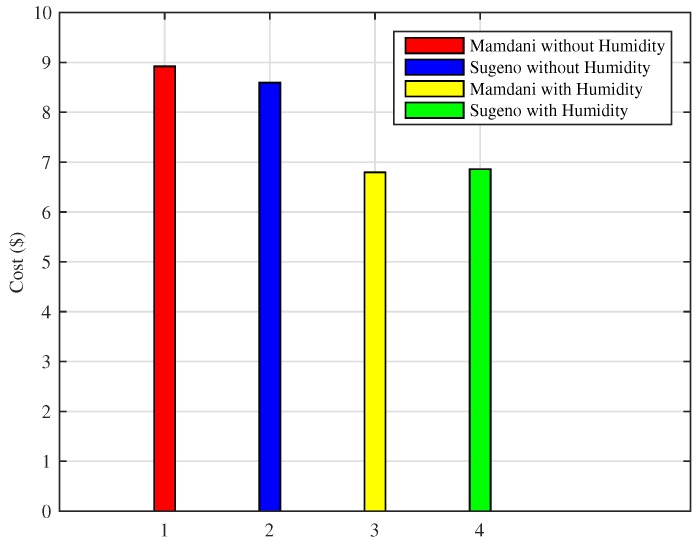
Daily cost for energy consumption in the hot cities.

Energy consumption minimization has the inevitable consequence of bill reduction. The cost for hot cities is calculated as using Equation (3). Figure 23 shows that proposed FLC performs better as compared to existing FLC for one day. Using the technique of Mamdani FIS without humidity, the cost is nearly $8.92, Sugeno FIS without humidity costs $8.59, Mamdani FIS with humidity approach costs $6.79 and Sugeno FIS with humidity FIS costs $6.86. The proposed technique of FLC using Mamdani FIS with humidity reduces the cost by 23.87% and Sugeno FIS with humidity shows efficiency of 23.09% as compared to the existing FLC without humidity as an input parameter. Mamdani FIS outperforms here because of its simple nature and results in bill reduction.

**Figure 24:**
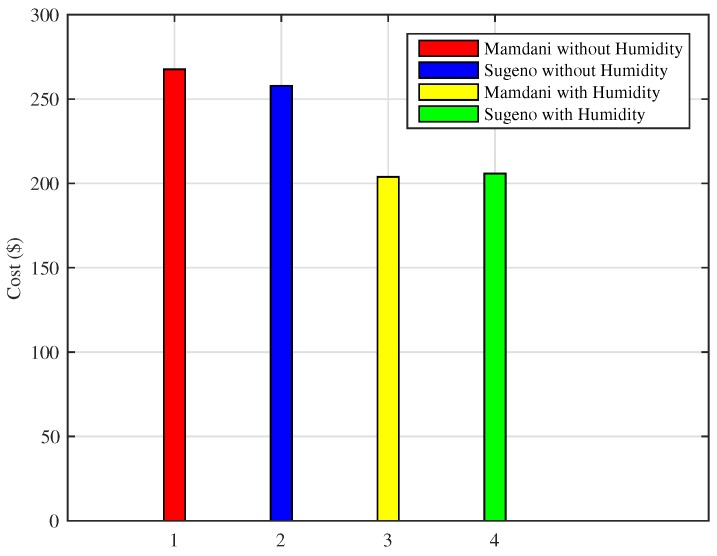
Monthly cost of energy consumption in the hot cities.

A similar conclusion is reached when monthly expense of using HVAC is calculated as shown in Figure 24. Mamdani FIS with humidity as an input parameter reduced the energy consumption resulting in a total cost of $203.83 in a month. Sugeno FIS with humidity as an environmental variable results in a total cost of $205.80 for a month. The total expenses incurred using proposed FIS are less as compared to existing FIS where Mamdani FIS costs $277.63 and Sugeno FIS without humidity parameter costs around $257.82 for a month.

4 Total Cost incurred with proposed FLC in Cold Cities

**Figure 25:**
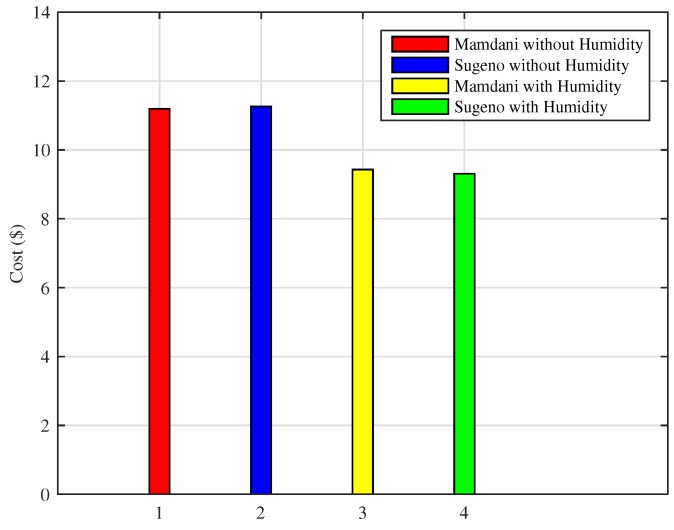
Daily cost for energy consumption in the cold cities.

**Figure 26:**
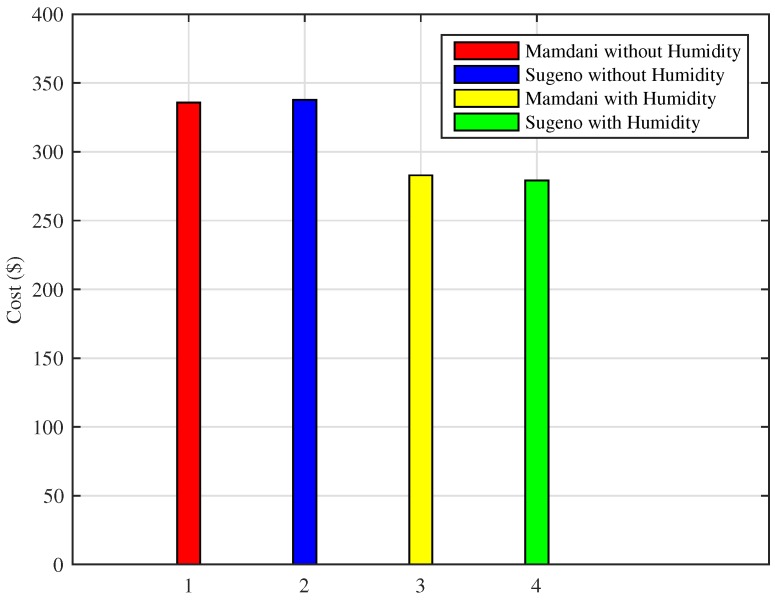
Monthly cost of energy consumption in the cold cities

As shown in Figure 25, the scheme using Mamdani FIS without humidity results in total cost of $11.19, Sugeno FIS without humidity costs $11.25, whereas the proposed Mamdani FIS with humidity shows that total cost incurred is $9.42, and Sugeno FIS with humidity costs $9.30 for energy consumption in a day. Moreover, Mamdani FIS with humidity shows the efficiency of 16.44% in cost reduction and 17.33% efficiency of Sugeno FIS with humidity in bill cost reduction as compared to the existing FIS without including humidity as an input parameter. We also compared the result of monthly consumption using proposed techniques for the cold city with those of the existing methods. As shown in Figure 26, Mamdani FIS and Sugeno FIS with humidity variables result in monthly costs of $282.87 and $279.17, respectively. These results show an increase in efficiency as the Mamdani FIS without humidity and Sugeno FIS without humidity as an input parameter results in the monthly cost of $335.75 and $337.76, respectively.

5 Results for PAR using proposed FIS

**Figure 27:**
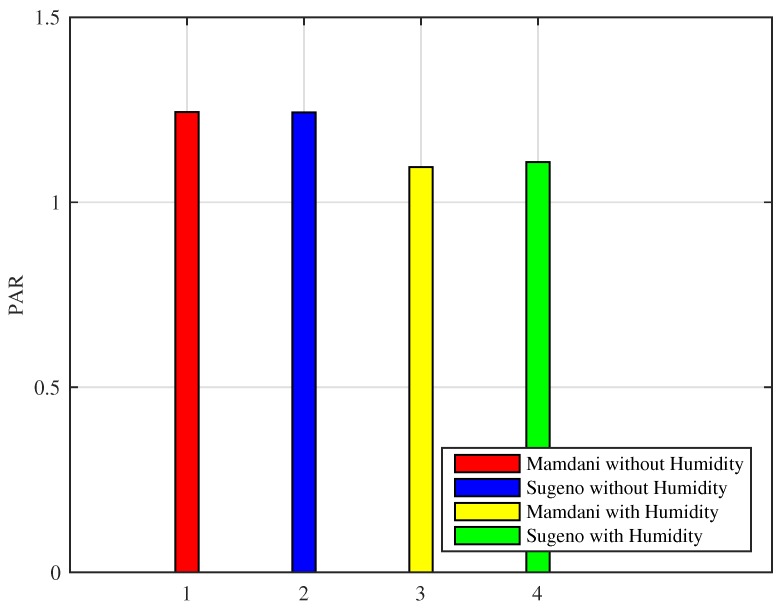
Peak-to-Average Ratio (PAR) of cold cities.

Peak-to-Average Ratio (PAR) is an important measure that is compared and observed when energy consumption is minimized either by load shifting or by load curtailment. It is often seen that appliance scheduling often results in an increase in the PAR when shifting load from high peak to low peak hours. PAR of the cold cities is shown in Figure 27. Clearly, it can be seen that Mamdani FIS with humidity achieved 12% efficiency as compared to Mamdani FIS without humidity, whereas the efficiency of Sugeno FIS with humidity is 10%. However, if the simulation was run for hot cities, no prominent efficiency was achieved. The reason behind this kind of behavior of proposed FLC is that it is mainly focused on the energy consumption minimization. Improvement in the PAR for cold cities can be considered as a by-product of the proposed FLC.

6 User Comfort maintenance using proposed FIS

The proposed FLC was working on the initialized set-points keeping in mind Figure 8, which shows a range of temperature that lies in user comfort for a particular relative humidity value. It was observed that, in previous techniques like [28,44], user comfort was mostly sacrificed. Using Figure 8, we are allowed to set high set-points for hot cities and low set-points for cold cities. Thus, it can be said that having information of humidity not only results in energy consumption minimization, but it also helps in maintaining the user comfort.

## 6. Conclusions

On the basis of the results, we have concluded that, by adding a feedback loop to the FIS with the input parameter of indoor temperature, outdoor temperature, occupancy, set-points, and price tariff, energy consumption required for a small room with a heater can be minimized as compared to previous techniques. Simulation results validated that the proposed method can reduce the energy consumption up to 50% in the worst case scenario.

There are several different parameters that can be considered to evaluate the user thermal comfort e.g., temperature, humidity, metabolic rate, etc. Different sensors are available that can be used to monitor environmental parameters like temperature, humidity, and occupancy. In order to enhance the user thermal comfort and minimize the energy consumption, a humidity parameter was integrated to the existing FIS. Rules in the FIS are designed on the combination of the input parameter’s membership function. Increasing the parameter increases the number of rules to be defined in the fuzzy rule base. In order to tackle different kinds of scenarios, defining more rules is beneficial. A method based on combinatorics was also defined, which assigns consequent to the antecedents of the fuzzy rules based on human intuition. MATLAB simulations were performed for one month that provides the validity of the proposed technique to reduce the energy consumption up to 28%. The proposed technique also maintained the user comfort while achieving electricity cost reduction up to 24%.

By comparing the results of two different and well-recognized fuzzy inference systems, it can be concluded that Mamdani FIS with humidity outperforms the existing techniques in the hot cities, whereas Sugeno FIS is more efficient in the cold city scenario when the humidity parameter is added. The main reason behind the difference in the performance of a proposed fuzzy inference system is the working nature of these systems. Comparative to the hot cities, cold city scenarios are more complex, which makes it more compatible with the complex nature of Sugeno FIS. In hot cities, Mamdani outperforms because of its simplest nature.

With the use of intelligent sensors and smart appliances, the proposed system is also capable of being implemented in IoT operating systems. The FIS proposed in this paper requires less memory, low processing power and real-time working capabilities. The proposed technique of FIS is able to be integrated in the RIOT, which is an operating system of networked IoT, mainly focusing on the low power devices with memory constraints.

Possible further improvement of the proposed research can be the inclusion of more input parameters to take into account the real scenarios in order to decide how much energy consumption is required. As the system proposed only deals with HVAC, possible extension to include more house appliances is also possible in order to save energy and maintain user comfort.

Enhancing the proposed system to work autonomously, which takes the information of the temperature and humidity from the sensor and decides the thermostat set-points automatically without user interaction is desirable for future work. Another possible extension to this system is to make the system adaptive according to user schedule. Because of global warming and depletion of fossil fuels, renewable energy sources are finding their way to the energy management system. This work can be further expanded to consider the potential effects of the renewable energy sources.

This system can also be expanded by applying the same fuzzy controller to a large number of houses and can be integrated into a micro grid. As the amount of data increases when dealing with a large number of homes, this system can be further integrated with cloud computing or fog computing. Big data analysis is used to improve the scalability of the smart grid component and can be added to the existing technique because of an increase in the system data when dealing with a large number of residential buildings. As the proposed system has the capability to be implemented in the IoT operating system, future studies should aim to for development of the system on a IoT networked platform.

## Figures and Tables

**Table 1:** Nomenclature.

Variables and Abbreviations	Description	Variables and Abbreviations	Description
FIS	Fuzzy Inference System	HEMS	Home Energy Management System
HVAC	Heating, Ventilation and Air Conditioning	PAR	Peak-to-Average Ratio
PCT	Programmable Communicating Thermostat	ToU	Time of Use
$Temp_{indoor}$	Indoor temperature fuzzy input parameter	$Temp_{outdoor}$	Outdoor temperature fuzzy input parameter
Occ	Occupancy fuzzy input parameter	$P_{rates}$	Electricity rate fuzzy input parameter
$ISP_{s}$	Thermostat set point fuzzy input parameter	$Humidity_{rel}$	Relative humidity fuzzy input parameter

**Table 2:** Summary of the previous scheduling techniques and fuzzy logic system.

Reference	Technique	The objective	A limitation
An efficient power scheduling scheme [30]	Hybrid of Knapsack Problem (K-WDO)	Minimization of the appliance waiting time and electricity cost.	Thermal comfort is ignored.
Air-conditioning system for proactive power demand response [32]	DSB and DFR	Cost and energy saving.	Use of synthetic dynamic prices and the system only works for offices.
The smart thermostat: using occupancy sensors [37]	Hidden Markov Model for occupancy based scheduling	Improved energy conservation.	Thermal comfort is sacrificed. Simulations are limited to only one type of HVAC.
Occupancy behavior-based model predictive control [39]	Occupancy based Model Predictive Control (MPC)	User comfort enhancement and energy consumption minimization.	High computational cost and increase the complexity of the system.
Hybrid Bacterial Foraging and Genetic Algorithm Optimization Techniques [40]	Hybrid of BFA and GA	Reduction in cost and PAR.	Thermal comfort is neglected. Only one pricing scheme is considered.
Scheduling Appliances with GA, TLBO, FA, OSR and Their Hybrids [41]	Hybrid of OSR with TLBO, FA, GA	Reduction in appliance waiting time, cost, PAR, and energy consumption.	Limited number of appliances. HVAC is not considered.
Dynamic demand response controller based on RTP [42]	Dynamic Demand Response Controller (DDRC)	Energy consumption minimization	Narrow range of the temperature band is considered. User preference is ignored.
A fuzzy logic system for demand-side load management [43]	Fuzzy logic rule based algorithm	Demand response participation. Minimization of the energy consumption.	User comfort is sacrificed.
An autonomous system via fuzzy logic [44]	Autonomous thermostat with Fuzzy Logic System	Energy conservation	Region-specific study. Only Mamdani FIS is considered.
An adaptive fuzzy logic system [17]	Adaptive Fuzzy Logic Model (AFLM)	Adapt the thermostat set-points according to user comfort. Energy consumption minimization.	The proposed technique only considered the cold regions. User comfort is heavily disturbed.
Worldwide adaptive thermostat using fuzzy inference system [28]	World-wide adaptive thermostat	Works for both cold and hot cities. Reduction in peak, cost and energy consumption.	User comfort is jeopardized.

**Table 3:** Variables used in Score calculation.

*i*	$v_{i}$
1	$Temp_{indoor}$
2	$Temp_{outdoor}$
3	Occ
4	$P_{rates}$
5	$ISP_{s}$
6	$Humidity_{rel}$

**Table 4:** Sample of rules defined in the proposed Fuzzy Inference System rule base.

#Rule	$T_{\mathrm{in}}$	$T_{\mathrm{out}}$	Rate	Occupant	ISP	Humidity	EC
1	L	L	HP	A	L	L	VL
2	L	M	OP	P	L	L	M
3	L	H	MP	P	M	H	M
4	M	H	OP	A	H	H	H
5	M	L	MP	P	M	M	M
6	H	M	OP	A	L	M	M
7	H	H	OP	P	H	H	VH
